# News from Societies

**Published:** 1960-01

**Authors:** 


### *News from Societies*

### WEST OF ENGLAND CHILD HEALTH GROUP

*Secretary:* Dr. A. L. Smallwood, Department of Public Health, Tower Hill, Bristol 2

10th Dec., at 8.30 p.m.: M. B. Lennard, Esq., M.V., CH.B., **"Family Practitioner Problems and Domiciliary Care".**

7th Jan., at 5.30 p.m.: Mrs. J. Madders, **"The Application of Physiotherapy in Special Needs".** In the Small Physics Lecture Theatre at the Royal Fort.

18th Feb., at 5.30 p.m.: H. C. Trowell, Esq., O.B.E., M.D., F.R.C.P., **"Paediatrics in the Tropics".**

10th Mar., at 8.30 p.m.: Miss Suzanne K. R. Clarke, M.D., **"Delinquency among Orphan Viruses".**

7th May, Visit to the Cotswold School, Ashton Keynes, nr. Swindon: C. A. Joyce, Esq., M.B.E., M.A., Headmaster.

Meetings are held in the Department of Child Health Lecture Theatre at the Children's Hospital, unless otherwise stated.

### B.M.A. (Barnstaple Division)

*Secretary:* Dr. S. G. Brook, Eddleston, Park Lane, Barnstaple

Dec. (date to be arranged): **Clinical Meeting** at the North Devon Infirmary in conjunction with the South-Western Branch.

20th Jan., at 8.30 p.m.: Professor C. Bruce Perry, M.D., F.R.C.P., **"Iatrogenic Disease, or the Hazards of Modern Therapy".** At the North Devon Infirmary.

### PLYMOUTH MEDICAL SOCIETY

*Secretaries:* Dr. Hugh Jolly, Yennadon House, Yelverton, and
Dr. Bernard Peck, 5 Lockington Ave., Hartley, Plymouth

11th Dec., at 8.30 p.m.: **Clinical Evening** at the Royal Naval Hospital.

15th Jan., at 8.30 p.m.: Robert V. Cooke, Esq., CH.M., F.R.C.S., **"Bees in My Bonnet"**, at Freedom Fields.

5th Feb., at 8.30 p.m.: **Clinical Evening** at Freedom Fields.

19th Feb., at 8.30 p.m.: **"Medicine and the Law".** Joint discussion meeting with the Incorporated Law Society of Plymouth. At Freedom Fields.

4th Mar., at 8.30 p.m.: P. D. Trevor-Roper, Esq., M.A., F.R.C.S., **"The Influence of Eye Disease on the Artists".** At Freedom Fields.

13th April, at 2.30 p.m.: Visit to Plymouth City Water Works.

9th May, at 8.30 p.m.: **Annual General Meeting** at Greenbank.

